# Covid-19 in China: ten critical issues for intensive care medicine

**DOI:** 10.1186/s13054-020-02848-z

**Published:** 2020-03-31

**Authors:** Li Li, Shijin Gong, Jing Yan

**Affiliations:** Department of Critical Care Medicine, Zhejiang Hospital, 12 Lingyin Road, Hangzhou, 310013 China

In December 2019, a newly identified coronavirus (SARS-CoV-2) major outbreak appeared in Wuhan City, Hubei Province, and it is now termed the Covid-19. The SARS-CoV-2 infection moved rapidly through China [1, 2] and spread to more than 90 countries. As of February 26, 2020, 78,064 patients were cumulative diagnosed, 12,224 cases were accumulative severe status. On January 29, 2020, the Chinese Society of Critical Care Medicine combined with the Chinese Medical Doctor Association of Critical Care Medicine and the Chinese Association of Pathophysiology of Intensive Care Medicine jointly issued a proposal to all of the Chinese intensive care colleagues to fight against Covid-19. According to the data from the National Health Commission of the People’s Republic of China, as of February 26, 2020, 29 provinces have dispatched 32,395 medical staff to support Wuhan City, of which 11,638 are intensive care physicians and nurses. Subsequently, more patients with Covid-19 have been effectively treated and the deaths of patients and the proportion of critically ill patients have shown a relatively declining trend in Wuhan. In fighting against Covid-19, intensive care physicians and nurses are not only the main force in the frontline, but also summarized and published valuable clinical study results in the first time, which provides useful first-hand clinical data for deepening the understanding of Covid-19, which mostly benefited from the rapid development of Chinese intensive care medicine in the past 20 years. More importantly, from this epidemic, we should find problems and sum up experiences. The following critical issues need to be concerned (Table 1).

**Table 1:** Ten critical issues for intensive care medicine

	Current situations or problems	Suggestions or solutions
1	Severe shortage of critical medical resources including physicians, nurses, and ICU beds in China.	Increase financial and staff investment, value the training of intensive care physicians and intensivists, and increase the ratio of ICU beds to hospital beds nationwide.
2	The levels of intensive care medicine in different provinces are uneven.	Strengthen training to achieve the homogeneity of clinical cognition and management capabilities of intensive care physicians in different provinces.
3	To focus medical resources on rescuing large numbers of Covid-19 patients, many non-Covid-19 patients are unable to receive effective treatment.	Establish a professional agency for the integration and optimization of the allocation of critical medical resources.
4	Medical staffs in Wuhan City from other provinces or cities were unfamiliar with the local conditions and the early phage of rescuing was in unordered states.	Set up standardized operating procedures for ensuring the refined risk stratification and subsequent refined management of critically ill patients.
5	Lack protection awareness and equipment early, many physicians or nurses were infected by SARS-CoV-2.	Strengthen occupational protection training and develop special standardized protection procedures during epidemics.
6	The continuous recognition of Covid-19 by Chinese intensive care physicians is a long and deepening process.	Develop the ability of intensive care physicians to quickly analyze and respond to a new disease.
7	Covid-19 patients usually are complicated with multiple organ failure.	More cooperation between intensive care medicine and other disciplines to deal with the rapid development of Covid-19.
8	The timing, dose, and duration of many therapies or life support for Covid-19 still remain controversial.	More training to improve the research ability of intensive care physicians.
9	The published studies mostly are single-centered and retrospective. It is difficult to integrate clinical, basic and public health data.	Establish a national special disease database, specimen bank and share research data from different medical centers.
10	Critical patients obtain multi-disciplinary expert team consultations through a national video remote consultation platform during Covid-19.	Promote ICU informatization construction. The successful experience should be extensively extended to the cure of other critical patients.

First, the proportion of the critical care unit (ICU) beds is seriously insufficient in China. According to the survey data from the Chinese Society of Critical Care Medicine, the ratio of ICU beds to hospital beds nationwide is only 1.65%, which means that there are only 3.43 ICU beds per 100,000 people. Certainly, when epidemic outbreaks such a large number of critically ill patients are huge challenges for any province or city.

Second, there is an urgent need to strengthen training to achieve the homogeneity of clinical cognition and management capabilities of intensive care physicians. From 2009 to 2019, 24,639 physicians were certified as intensive care physicians in the Chinese Critical Care Certified Course (5C) program in China, only accounting for about 30% of physicians engaged in intensive care medicine in China. The levels of intensive care medicine in different provinces are uneven. The top province in the number of participants is Jiangsu Province (1808, 9.55%), while Hubei Province only 610 participants (3.2%).

Third, professional agency needs to be established for the integration and optimization of the allocation of critical medical resources. During epidemic, not only to ensure the therapy of patients with severe Covid-19, but also to ensure that other critically ill patients, non-Covid-19 patients can also be effectively treated.

Fourth, standardized operating procedures should be set up for ensuring the refined risk stratification and subsequent refined management of critically ill patients. According to the severity of patients, not only the patients are allocated in a hierarchical manner, but also the allocation of critical medical resources from other provinces and cities.

Fifth, it is necessary to strengthen occupational protection training and develop special standardized protection procedures for high-risk invasive operations during epidemics. In Hubei Province, more than 3000 physicians or nurses were infected by SARS-CoV-2. To reduce the operation or exposure time it might be necessary to set up special operation teams such as extracorporeal membrane oxygenation (ECMO) team and continuous renal replacement therapy (CRRT) team*.*

Sixth, the ability of intensive care physicians to quickly analyze and respond to new disease needs to be developed. China has experienced seven versions of consensus for Covid-19 diagnosis and treatment. In the beginning, most intensive care physicians believed that SARS-CoV-2 infection mainly induced viral pneumonia, and applied symptomatic treatment mainly targeted at the respiratory system. It was later discovered that deaths were often accompanied by multiple organ failure and cytokine storm syndrome (CSS), which are important causes of sudden exacerbations and death [3, 4]. Passing through the above course is the process of continuous recognition of SARS-CoV-2 infected pneumonia by Chinese intensive care physicians, which is based on large numbers of patients with critical illness or even death.

Seventh, intensive care medicine requires more cooperation with multi-disciplines. The main target of SARS-CoV-2 infection in humans is the angiotensin-converting enzyme 2 (ACE2) genes. In addition to the alveoli, the ACE2 gene is highly expressed in the heart, kidney, and small intestine, which means that the virus may also infect these organs [5]. Multi-disciplines team should integrate the infectious diseases, respiratory, nephrology, intensive care, and pathology physicians to deal with the rapid development of Covid-19.

Eighth, the research ability of intensive care physicians needs to be improved. What is the mechanism of the drastic reduction of lymphocytes in patients with poor prognosis? Could the patients’ prognosis be improved by enhancing immune function? When does tracheal intubation, invasive mechanical ventilation, or ECMO support be used to most effectively protect organ functions and reduce mortality? [6] In addition, the timing, dose, and duration of systemic corticosteroids still remain controversial.

Ninth, it is urgent to establish a special disease database, specimen bank, and share research data from different medical centers. So far, the published articles mostly are from single-centered and retrospective studies. It is difficult to integrate clinical, basic, and public health data, which makes it unnecessary for intensive care medicine to play a leading role in rescuing Covid-19.

Tenth, it is necessary to promote ICU informatization construction and establish the national remote consultation platform through 4G or 5G wireless networks. During Covid-19, video remote consultation has constructed to ensure that critically ill patients obtain timely multi-disciplinary expert team consultations. This successful experience should be extensively extended to the cure of other critical patients.

In summary, during the fight against Covid-19, we need to think and discover problems in the development of intensive care medicine, find the critical issues to be improved and ultimately promote the development of intensive care medicine.

## Data Availability

Not applicable.
